# Evaluation of long-term consumption of omeprazole disadvantages: a network analysis

**Published:** 2020

**Authors:** Abdolrahim Nikzamir, Mostafa Rezaei-Tavirani, Mohhamadreza Razzaghi, Sina Rezaei Tavirani, Maryam Hamzeloo-Moghadam, Somayeh Esmaeili, Behzad Hatami, Alireza Ahmadzadeh

**Affiliations:** 1 *Faculty of Medicine, Shahid Beheshti University of Medical Sciences, Tehran, Iran *; 2 *Proteomics Research Center, Faculty of Paramedical Sciences, Shahid Beheshti University of Medical Sciences, Tehran, Iran*; 3 *Laser Application in Medical Sciences Research Center, Shahid Beheshti University of Medical Sciences, Tehran, Iran*; 4 *Gastroenterology and Liver Diseases Research Center, Research Institute for Gastroenterology and Liver Diseases, Shahid Beheshti University of Medical Sciences, Tehran, Iran*; 5 *Traditional Medicine and Materia Medica Research Center and Department of Traditional Pharmacy, School of Traditional Medicine, Shahid Beheshti University of Medical Sciences, Tehran, Iran *

**Keywords:** Omeprazole, Long term consumption, Gene expression

## Abstract

**Aim:**

Evaluation of deregulated genes after long-term consuming of omeprazole via network analysis.

**Background:**

Proton pump inhibitors (PPIs) are used to inhibit gastric high rate of acid secretion in patients. Omeprazole as a PPI is a common drug in this regard. Evaluation of long-term consumption of omeprazole is studied in the present study via its effects on the gene expression of “human coronary artery endothelial cells”.

**Methods:**

Net effect of the presence of omeprazole on gene expression profiles of “human coronary artery endothelial cells” was evaluated through data from gene expression omnibus (GEO). Results of protein-protein interaction (PPI) network analysis were assessed via biological process examination to find the critical deregulated genes after long-term consumption of omeprazole.

**Results:**

“Negative regulation of muscle cell apoptotic process”, “negative regulation of DNA binding”, “telencephalon cell migration”, “forebrain cell migration” “response to cadmium ion”, “cell-cell recognition”, “positive regulation of protein targeting to mitochondrion”, and “central nervous system neuron development” were the clusters of biological processes that were associated to the long -term presence of omeprazole. The final critical deregulated genes were JAK2, PTK2, and NRG1.

**Conclusion:**

It can be concluded that cell cycle, proliferation, and apoptosis and several essential biological processes are affected and nervous system is a possible target related to the long-term consumption of omeprazole.

## Introduction

 In biology and medicine, network analysis is a suitable method to screen large data to find the critical ones as biomarkers (1). In this approach, the interacted genes or biomacromolecules are analyzed to find the crucial individuals (2). Centrality analysis based on the properties of connections between the nodes of the network provides valuable information about the interrelationship of the elements of the network (3). Degree is a centrality parameter that refers to the numbers of first neighbors of a node (4). The nodes with a high value of degree are known as hubs. Hub nodes are important key elements of network employed in the studied systems (5). The other important centrality parameters is betweenness centrality that is a function of the shortest paths passing through the node (6). The nodes which are characterized with a high value of betweenness are identified as bottlenecks (7). Bottleneck nodes are considered as essential elements of the analyzed network (8). The common nodes between hubs and bottlenecks are well-known as hub-bottlenecks, regarded as key nodes of the network (9). There are other central parameters such as closeness and stress that are used to discriminate the nodes of the deliberated networks (10). Biological process as a component of gene ontology is utilized to screen the genes. Molecular mechanism of several diseases are investigated via biological process analysis (11). 

In the present study, the deregulated genes after long-term consuming of omeprazole are evaluated via network analysis to find the critical genes through centrality parameters. The critical deregulated genes are investigated by biological process analysis and the key genes are introduced that are associated with the long-term consumption of omeprazole. 

## Methods

Data is obtained from Costarelli et al. record in Gene Expression Omnibus (GEO) database entitled GSE77239. As it is described by the author’s gene profiles of senescent and non-senescent human coronary artery endothelial cells (HCAECs) in the presence and absence of omeprazole are compared by cDNA microarray (12). The gene expression profiles of four sets including young and old samples in the absence and presence of omeprazole were matched statistically via box plot analysis by GEO2R. Top 250 DEGs based on p-values were downloaded for young-old comparison in the absence and presence of omeprazole separately. The characterized significant DEGs based on p-value<0.01 and fold change ≥ 4 were determined for more investigations. The significant DEGs associated with the analysis in the presence of omeprazole which were not common with the analysis in the absence of omeprazole were identified as significant DEGs associated with long-term consumption of omeprazole. 

The identified significant DEGs were included in a PPI network via STRING database (13) by Cytoscape software (14). To maximize interactions between the queried DEGs, 30 first neighbor genes from STRING database were added to the significant DEGs and the network was constructed. The main connected component of the network was analyzed by Network analyzer; an application of Cytoscape software. Four centrality parameters of degree, betweenness centrality, closeness centrality, and stress were determined for all nodes of the main connected component. The top 10 nodes (among the queried DEGs) based on each centrality parameters were strongminded as central nodes. In this regard, 40 central nodes that were common between all four groups were firmed as critical central nodes.

The biological processes that were associated with 104 significant DEGs were recognized and clustered from GO-biological process via ClueGO (15) plugin of Cytoscape software. Gene ontology (GO) term p-value, term p-value corrected with Bonferroni step down, group p-value, and group p-value corrected with Bonferroni step down <0.05 were considered. Action map including activation, inhibition, and expression was done via CluePedia (16). The genes that were involved in the determined biological processes were analyzed based on participation rate in related biological processes. 

## Results

**Figure 1:**
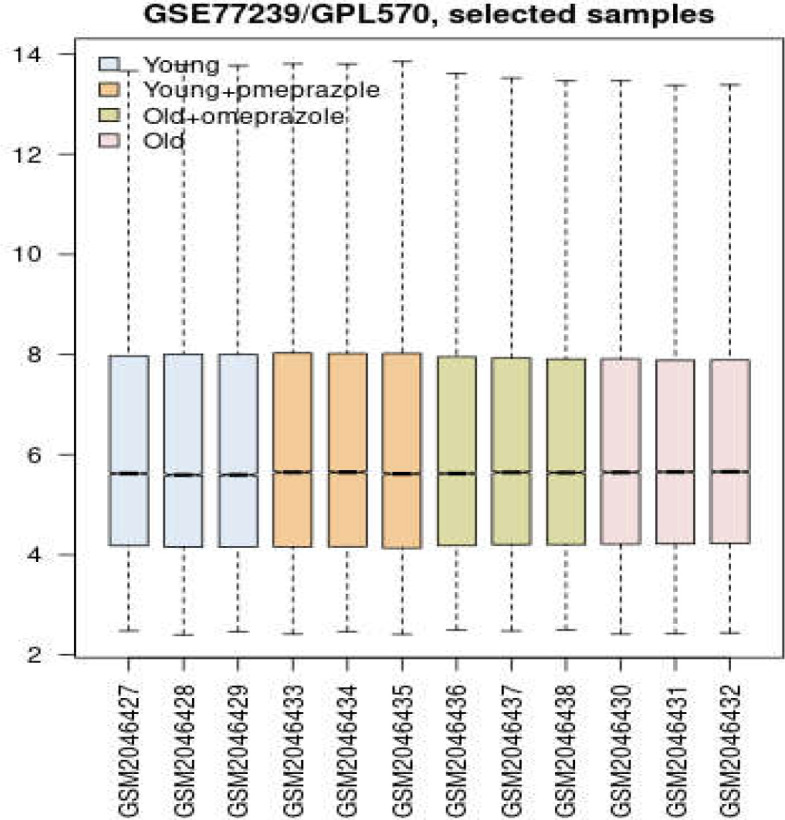
Box plot analysis for gene expression profiles of the four sets of cultured HCAECs

Results of box plot analysis for gene expression profiles of the four groups of culture cells is presented in figure 1. 

**Figure 2:**
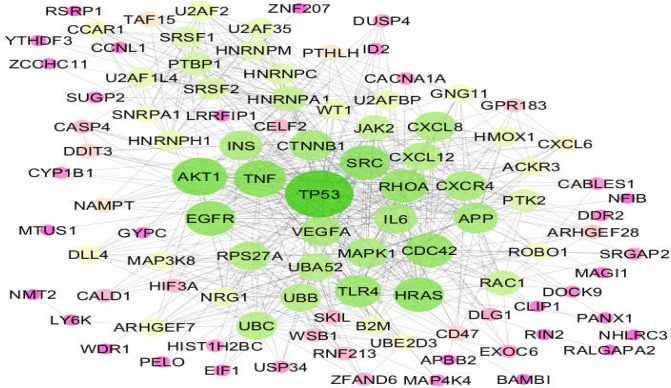
The main connected component of the PPI network including 71 queried DEGs and 30 added first neighbor genes. The node are connected by 664 links. Larger size of nodes is associated with larger amounts of degree value

**Figure 3:**
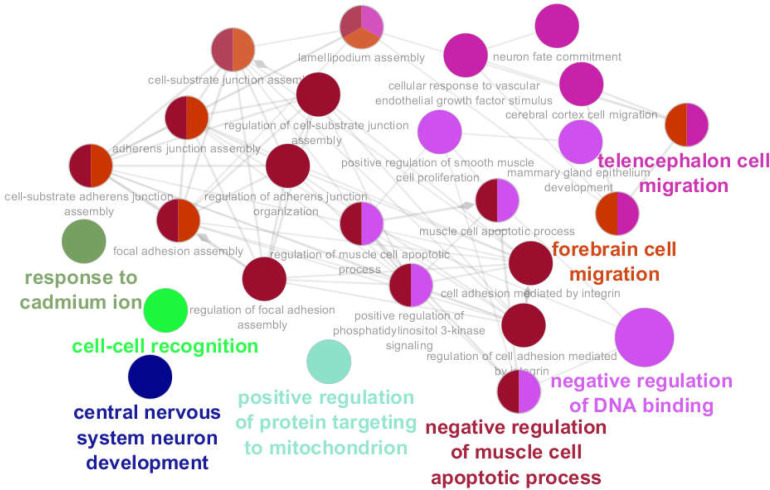
Eight clusters including 26 biological processes which are associated with the 104 significant DEGs are presented. The terms that are shown in 2 colors are common between the 2 clusters

**Figure 4:**
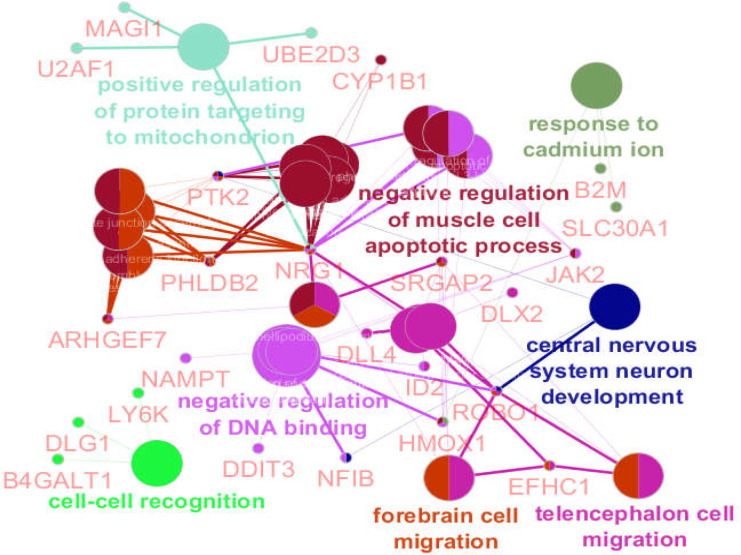
The genes and the related clusters of biological processes are shown. There were not any activation, inhibition, and expression actions between genes. Some genes are connected to more than one cluster

**Table 1:** Critical central nodes are presented. Descriptions are obtained from STRING database and summarized. K, BC, and CC correspond to degree, betweenness centrality, and closeness centrality respectively

R	Gene	K		BC	CC	Stress	description
1	HNRNPA1	23		0.045	0.510	4256	Heterogeneous nuclear ribonucleoprotein A1 involved in the packaging of pre-mRNA into hnRNP particles, transport of poly(A) mRNA from the nucleus to the cytoplasm and possibly modulating splice site selection, and binding to specific miRNA hairpins.
2	JAK2	23		0.012	0.518	1494	Tyrosine-protein kinase JAK2; Non-receptor tyrosine kinase involved in processes such as cell cycle, cell growth, development, differentiation or histone modifications. Mediates essential signaling events in both innate and adaptive immunity. In the cytoplasm, plays a pivotal role in signal transduction via its association with type I receptors such as growth hormone (GHR), prolactin (PRLR), leptin (LEPR), erythropoietin (EPOR), thrombopoietin (THPO); or type II receptors including IFN-alpha, IFN-beta, IFN-gamma and multiple interleukins. Is involved in cytokine-driven activation of FOS transcription. Plays a key role in chromatin in the nucleus.
3	PTK2	19		0.004	0.495	534	Protein phosphatase 1 regulatory subunit 71; plays an essential role in regulating cell migration, adhesion, spreading, reorganization of the actin cytoskeleton, formation and disassembly of focal adhesions and cell protrusions, cell cycle progression, cell proliferation and apoptosis. Required for early embryonic development and placenta development. Required for embryonic angiogenesis, normal cardiomyocyte migration and proliferation, and normal heart development. Regulates axon growth and neuronal cell migration, axon branching and synapse formation; required for normal development of the nervous system. Plays a role in osteogenesis and differentiation of osteoblasts
4	WT1	15		0.008	0.478	1086	Wilms tumor protein; Transcription factor that plays an important role in cellular development and cell survival. Regulates the expression of numerous target genes, including EPO. Plays an essential role for the development of the urogenital system. Has a tumor suppressor as well as an oncogenic role in tumor formation.
5	B2M	14		0.009	0.500	742	Beta-2-microglobulin; Component of the class I major histocompatibility complex (MHC). Involved in the presentation of peptide antigens to the immune system. Exogenously applied M.tuberculosis EsxA or EsxA-EsxB (or EsxA expressed in host) binds to B2M and decreases its export to the cell surface (total protein levels do not change), probably leading to defects in class I antigen presentation.

**Table 2:** List of the 8 clusters of biological processes is presented. The bold term is the name of cluster and Gog, and %AG and Nr.G refer to gene ontology group and numbers of gene, respectively. Gene ontology (GO) term p-value, term p-value corrected with Bonferroni step down, group p-value, and group p-value corrected with Bonferroni step down <0.05 were considered

R	GOTerm	GOg	% A G	Nr. G	Associated Genes Found
1	**response to cadmium ion**	1	4.55	3	[B2M, HMOX1, SLC30A1]
2	**cell-cell recognition**	2	4.11	3	[B4GALT1, DLG1, LY6K]
3	**positive regulation of protein targeting to mitochondrion**	3	4.30	4	[MAGI1, NRG1, U2AF1, UBE2D3]
4	**central nervous system neuron development**	4	4.05	3	[NFIB, PTK2, ROBO1]
5	lamellipodium assembly	5	4.76	3	[ARHGEF7, NRG1, SRGAP2]
6	cellular response to vascular endothelial growth factor stimulus	5	6.25	3	[DLL4, NRG1, ROBO1]
7	neuron fate commitment	5	5.80	4	[DLL4, DLX2, ID2, NRG1]
8	**forebrain cell migration**	5	5.97	4	[EFHC1, NRG1, ROBO1, SRGAP2]
9	telencephalon cell migration	5	6.25	4	[EFHC1, NRG1, ROBO1, SRGAP2]
10	cerebral cortex cell migration	5	6.12	3	[EFHC1, ROBO1, SRGAP2]
11	cell-substrate junction assembly	6	4.08	4	[ARHGEF7, NRG1, PHLDB2, PTK2]
12	adherens junction assembly	6	4.44	4	[ARHGEF7, NRG1, PHLDB2, PTK2]
13	lamellipodium assembly	6	4.76	3	[ARHGEF7, NRG1, SRGAP2]
14	cell-substrate adherens junction assembly	6	5.00	4	[ARHGEF7, NRG1, PHLDB2, PTK2]
15	forebrain cell migration	6	5.97	4	[EFHC1, NRG1, ROBO1, SRGAP2]
16	focal adhesion assembly	6	5.00	4	[ARHGEF7, NRG1, PHLDB2, PTK2]
17	**telencephalon cell migration**	6	6.25	4	[EFHC1, NRG1, ROBO1, SRGAP2]
18	positive regulation of smooth muscle cell proliferation	7	5.26	4	[HMOX1, ID2, JAK2, NAMPT]
19	muscle cell apoptotic process	7	5.26	3	[HMOX1, JAK2, NRG1]
20	**negative regulation of DNA binding**	7	7.41	4	[DDIT3, HMOX1, JAK2, NFIB]
21	regulation of muscle cell apoptotic process	7	5.66	3	[HMOX1, JAK2, NRG1]
22	mammary gland epithelium development	7	4.23	3	[ID2, JAK2, ROBO1]
23	negative regulation of muscle cell apoptotic process	7	9.09	3	[HMOX1, JAK2, NRG1]
24	positive regulation of phosphatidylinositol 3-kinase signaling	7	4.23	3	[JAK2, NRG1, PTK2]
25	cell adhesion mediated by integrin	8	5.08	3	[CYP1B1, NRG1, PTK2]
26	regulation of cell adhesion mediated by integrin	8	7.32	3	[CYP1B1, NRG1, PTK2]
27	cell-substrate junction assembly	8	4.08	4	[ARHGEF7, NRG1, PHLDB2, PTK2]
28	muscle cell apoptotic process	8	5.26	3	[HMOX1, JAK2, NRG1]
29	regulation of cell-substrate junction assembly	8	4.92	3	[NRG1, PHLDB2, PTK2]
30	regulation of adherens junction organization	8	4.69	3	[NRG1, PHLDB2, PTK2]
31	adherens junction assembly	8	4.44	4	[ARHGEF7, NRG1, PHLDB2, PTK2]
32	lamellipodium assembly	8	4.76	3	[ARHGEF7, NRG1, SRGAP2]
33	cell-substrate adherens junction assembly	8	5.00	4	[ARHGEF7, NRG1, PHLDB2, PTK2]
34	regulation of muscle cell apoptotic process	8	5.66	3	[HMOX1, JAK2, NRG1]
35	focal adhesion assembly	8	5.00	4	[ARHGEF7, NRG1, PHLDB2, PTK2]
36	**negative regulation of muscle cell apoptotic process**	8	9.09	3	[HMOX1, JAK2, NRG1]
37	regulation of focal adhesion assembly	8	4.92	3	[NRG1, PHLDB2, PTK2]
38	positive regulation of phosphatidylinositol 3-kinase signaling	8	4.23	3	[JAK2, NRG1, PTK2]

As shown in this figure, Distribution of gene expression values are median centered so further analysis is possible. A total of 104 significant DEGs were identified as the genes related to the long-term effects of omeprazole consumption. The 104 Queried genes were included in the PPI network where 97 individuals were recognized be STRING database. The final network was formed by the 97 DEGs plus 30 added first neighbor genes. The network contained 22 isolates DEGs, 2 paired DEGs, and a main connected component including 101 nodes (71 GEGs and 30 added first neighbor genes). The visualized main connected component of network based on degree value is presented in Figure 2. Five critical central nodes including HNRNPA1, JAK2, PTK2, WT1, and B2M were highlighted. Except PTK2 that were common in the top 10 groups of degree betweenness centrality, and closeness centrality the other 4 individuals were common in all four groups. Central properties and descriptions of the critical nodes are shown in Table 1.

Eight clusters including 26 biological processes were recognized associated with significant DEGs (see table 2 and figure 3). “Negative regulation of muscle cell apoptotic process”, “negative regulation of DNA binding”, “telencephalon cell migration”, “forebrain cell migration” “response to cadmium ion”, “cell-cell recognition”, “positive regulation of protein targeting to mitochondrion”, and “central nervous system neuron development” were the recognized clusters. 

**Figure 5:**
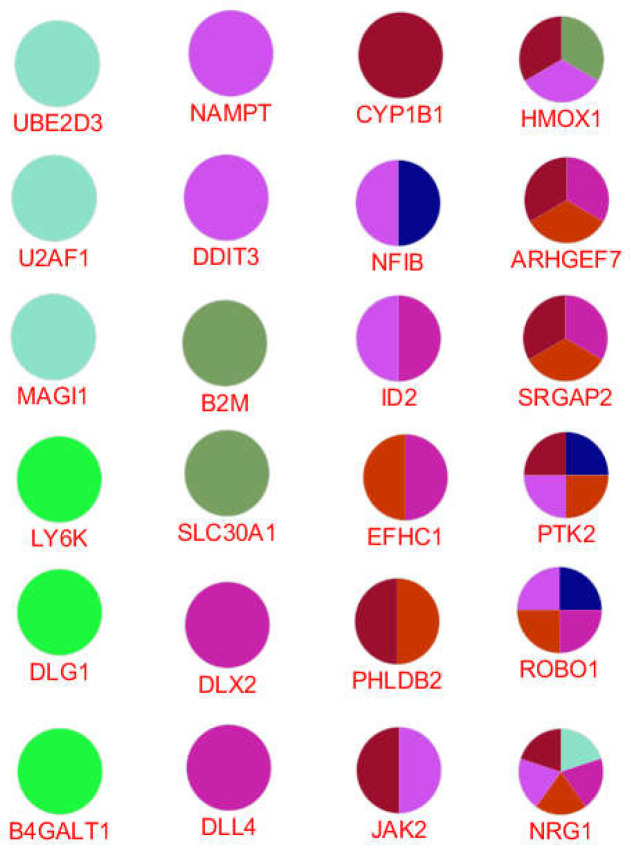
The 24 recognized DEGs that are related to the biological processes are presented. The genes that are related to one cluster are represented in columns 1 and 2. Column 3 refers to the genes that are involved in 2 clusters. The genes which are linked to 3, 4, and 5 clusters are shown in column 4. The presented colors correspond to the colors of clusters in Figure 4

As demonstrated in Figure 4, only 24 DEGs among the 104 queried genes were recognized with ClueGO. For better resolution, the 24 introduced genes are shown in Figure 5. As illustrated in Figure 5, 13, 5, 3, 2 and 1 genes are related to 1, 2, 3, 4, and 5 clusters of biological terms respectively. NRG1 is the only gene associated with 5 clusters.

## Discussion

Distributions of data are validated statistically and the introduced significant DEGs are screened by high values of fold change. It can be concluded that the selected genes discriminate long-term versus short-time consumption of omeprazole.

There are many references about the safety of omeprazole but it is reported recently that long-term treatment with omeprazole is accompanied with serious physiological consequences. As Yu-chen Yang et al. found long-term physiological and Microbiota changes in rats after the administration of omeprazole (17). In the present study, a large number of genes deregulated in the long-term presence of omeprazole were screened to find clear evidence of possible molecular events in the cardiovascular system. A total of 104 significant genes were candidate as DEGs. Two analytical methods including centrality analysis and gene ontology were considered to investigate the role of these DEGs. Based on the centrality analysis, 5 central DEGs including HNRNPA1, JAK2, PTK2, WT1, and B2M were determined as key deregulated genes. Except PTK2, the other 4 genes were selected among top genes based on four important centrality parameters of degree, betweenness centrality, closeness centrality and stress. 

Based on descriptions of the 5 central genes (see table 1), essential biological processes are associated with the function of these genes. Processes that are deregulated in the long-time presence of omeprazole are the following: RNA splicing, cell cycle, cell growth, development, differentiation and apoptosis, migration, adhesion, spreading or histone modifications, signaling events in both innate and adaptive immunity, function of several important hormones such as growth hormone, prolactin, leptin, erythropoietin, thrombopoietin, IFN-alpha, IFN-beta, IFN-gamma and multiple interleukins, cytokine-driven activation of FOS transcription, chromatin, reorganization of the actin cytoskeleton, formation and disassembly of focal adhesions and cell protrusions, early embryonic development and angiogenesis, early placenta development, normal cardiomyocyte migration and proliferation, normal heart development, axon growth and neuronal cell migration, axon branching and synapse formation, normal development of the nervous system, differentiation of osteoblasts, tumor suppressing and formation, and presentation of peptide antigens to the immune system. 

Gene ontology analysis led to identifying 24 genes among the queried 104 DEGs that were linked to 8 clusters of biological processes. As shown in table 2 and figure 3, there is a suitable overlap between the introduced biological processes and descriptions of the central genes. Three central genes of JAK2, PTK2, and B2M (60% of central DEGs) are presented among the 24 genes which were highlighted via gene ontology analysis. As it is represented in figure 5, PTK2 is connected to the 4 clusters of biological processes; “Negative regulation of muscle cell apoptotic process”, “negative regulation of DNA binding”, “forebrain cell migration”, and “central nervous system neuron development” are the recognized clusters. As it is shown in table 2, these 4 clusters include all biological processes. JAK2 is also linked to the two main clusters of biological processes. NRG1 is the highlighted gene that is connected to the 5 clusters of biological process but was not included in the central genes. It can be concluded that JAK2, PTK2, and NRG1 are the critical deregulated genes in the presence of long-time consumption of omeprazole.

Higher gastric cancer risk due to long-term treatment with proton pump inhibitors is investigated in several studies, but more investigation is required for more accurate results (18). Involvement of PKT2 mutation and promotion of several cancers are addressed in several studies (19, 20). In another investigation, they found a relationship between the long-term usage of proton pump inhibitors including omeprazole and the onset of both dementia and depression disorders (21). The recent finding corresponds with our finding: three clusters of biological processes are directly related to the nervous system. As a critical gene JAK2 plays a significant role in the function of nervous system. JAK2/STAT3 axis targeting is suggested in Alzheimer's disease (22). Reportedly, there is a relationship between JAK2 mutations and myeloproliferative diseases (23). studies indicate that NRG1 is involved in Hirschsprung's disease that is a congenital disorder with the absence of enteric ganglia in variable portions of the distal intestine (24). A possible relationship between the deregulation of NRG1 and schizophrenia is documented in a meta-analysis study (25).

Among a large number of deregulated genes, JAK2, PTK2, and NRG1 are regarded as critical genes associated with the long-term consumption of omeprazole. Essential biological processes such as cell cycle, proliferation, and apoptosis were highlighted as the affected terms. The analysis revealed that nervous system is a possible target related to the long-term usage of omeprazole.
